# Mutation accumulation is still potentially problematic, despite declining paternal age: a comment on Arslan *et al.* (2017)

**DOI:** 10.1098/rspb.2017.2511

**Published:** 2018-02-21

**Authors:** Michael A. Woodley of Menie, Matthew A. Sarraf, Heitor B. F. Fernandes

**Affiliations:** 1 Center Leo Apostel for Interdisciplinary Studies, Vrije Universiteit Brussel, Brussels, Belgium; 2 Unz Foundation Junior Fellow, Unz Foundation, Palo Alto, CA, USA; 3 University of Rochester, Rochester, NY, USA; 4 Department of Psychology, University of Arizona, Tucson, AZ, USA

Arslan *et al*. [1] found that paternal age negatively predicts offspring survival and reproductive success in four Western populations (three ‘historical’ and one ‘modern’, i.e. from the twentieth century) even after implementing controls for relevant covariates. The authors believe that this finding indicates that ‘purifying selection is still effective’ in at least one modernized population, and conclude that they ‘do not predict that contemporary reproductive timing will lead to unprecedented or unbearable de novo mutation loads and concomitant changes in the prevalence of genetic disorders' (p. 8), especially in light of the observation that average parental age has decreased over time, diminishing the number of deleterious de novo mutations added to the genomes of each new generation. We contend that negative selection acting on the relative fitness costs of mutations may be insufficient to prevent mutation accumulation in modernized populations and that the possibility of problematic increases in mutation load ought to be reconsidered.

Simulations indicate that considerably less reproductive failure (possibly as little as 20% per generation [2]) is needed to remove variants from populations under the assumption of selection based on relative fitness differentials (i.e. where the fitness costs of a given variant are estimated relative to the fitness of other genomes with different mutation loads) compared to absolute fitness differentials (i.e. where the fitness costs are estimated relative to a hypothetical mutation-free genome). On the assumption of absolute fitness differentials and given observed human de novo mutation rates, 88% of individuals must fail to reproduce, and an average fertility rate of 16 babies per woman in the reproducing fraction is required to purify a population of deleterious variants. This yields the *mutation load paradox*, or the incongruity between the high reproductive failure rate apparently required for population purification and the low observed reproductive failure rate in modernized populations [2].

Negative selection acting on relative fitness differentials may partly resolve the paradox; however, it may not be wholly adequate for this purpose. Estimates of the reproductive failure rate required for purification when negative selection acts on relative fitness are based on simulations [2] employing assumptions that may not be needed to make sense of negative selection in historical human populations. Infancy and childhood together constitute a potentially significant ‘vulnerable period’ in which deleterious mutations may strongly impact prospects for reproductive participation. This period has been termed ‘the crucible of human evolution’ [3] owing to the high levels of mortality during infancy and childhood that were the norm for the majority of human history (child mortality rates were as high as 50% in some parts of Europe during the Early Modern Era [3]).

If child and infant mortality were random with respect to deleterious variants, then they could not have been major sources of negative selection. There are reasons to doubt this possibility, however. First, mortality was non-random with respect to social status, with infants and children from lower-status families having had a far higher risk of death than those from higher-status ones [4]. Social status variation results from facultative calibration during development based on the action of heritable sources of cognitive and behavioural individual differences, which may be sensitive to deleterious mutations such that higher loads increase the risk of becoming low status. Furthermore, data from Medieval England indicate that low-status individuals typically produced children later in life than relatively higher-status ones [5], with social stress imposing fertility delays that likely increased the loads of de novo mutations among the former's offspring, increasing the chances of downward social mobility and reproductive failure. Second, the presence of the *general fitness factor*—a positive manifold among a large array of behavioural, cognitive and physiological indicators resulting from the pleiotropic action of deleterious mutations that reduce developmental stability [6]—suggests that in very competitive ecologies and in the absence of factors that would attenuate the fitness costs of mutations (such as advanced medicine), pleiotropic mutations may have been especially lethal due to their potential to impair functionality across a number of fitness-critical domains. Therefore, seemingly distinct selective pressures acting on different populations at different times historically may have in fact constituted negative selection acting on a single target—the general fitness factor. If this is the case, and the seemingly random environmental, health and social hardships that these pre- and early modern populations experienced *were* selecting for higher levels of general fitness, then the levels of reproductive failure in these populations were approaching those predicted by the *mutation load paradox*—chronically very high infant and child mortality rates throughout the great majority of human history having been necessary to purify historical populations of deleterious mutations. Therefore, the mutation load ‘paradox’ is not paradoxical because it would not apply to non-modernized, including premodern, populations, in which the optimal genome may have been virtually free of deleterious mutations.

The work of Henneberg [7] offers a comprehensive account of the role of early life mortality in influencing the opportunity for negative selection in various populations, as captured by his *Index of Biological State* (*I*_bs_)—a composite measure of various factors that reduce the potential for reproductive participation and thus increase the opportunity for negative selection. Scaled from 0 to 1 (where 1 = 100% probability that an average individual will get to fully participate in reproduction, and where the opportunity for negative selection is 0; [7]), historical data indicate that *I*_bs_ was very low for most of the last 15 000 years (*I*_bs_ values around 0.3–0.4). However, starting in the 1600s, *I*_bs_ values increased drastically (rising from approx. 0.5 to 0.99 in present-day Western populations) [8], potentially because reduced environmental harshness coupled with industrialization and innovations in hygiene and medicine significantly decreased the fitness costs of harbouring deleterious variants, allowing infant and child mortality to decline.

Paternal age will still predict reproductive success in modernized populations (as Arslan *et al*. [1] report), since even if the *absolute* fitness costs of many deleterious mutations have been attenuated, the *relative* fitness cost per unit of paternal age may be similar across populations over time, preserving the *rank order* in paternal age effects despite significantly reduced negative selection with respect to a large part of the mutation spectrum. However, with a far larger percentage of the overall population likely participating in reproduction, these mutations will accumulate across generations as part of a legacy load—persisting in populations for far longer than in the preindustrial era. Declining paternal age would not necessarily offset this trend either, given sufficiently relaxed negative selection. This is illustrated via reanalysis of data published by Kong *et al*. ([9], fig. 4, p. 474), who track the de novo mutation load of the population of Iceland over 3.5 centuries, using mean paternal age (which has been declining over time; *r* = −0.714, 95% CI = −0.882 to −0.385, *N* = 19 cohorts) as a proxy. Between the birth cohorts corresponding to the years 1650–1659 and 2010+, *I*_bs_ rose from 0.5 to 0.99 ([8], fig. 1, p. 3). By adding a cohort's load of de novo variants to the previous cohort's load multiplied by *I*_bs_, the effects of varying degrees of relaxed negative selection can be simulated. Allowing for a linear increase in *I*_bs_ across 3.5 centuries, the effects of changing strength of negative selection on legacy load additionally can be modelled. Here two simulations are run, one in which *I*_bs_ increases from 0.5 to 0.99 across 3.5 centuries, and a second in which *I*_bs_ is fixed to the value for most of the last 15 000 years (i.e. 0.35). The results are graphed in figure 1.

**Figure 1.**
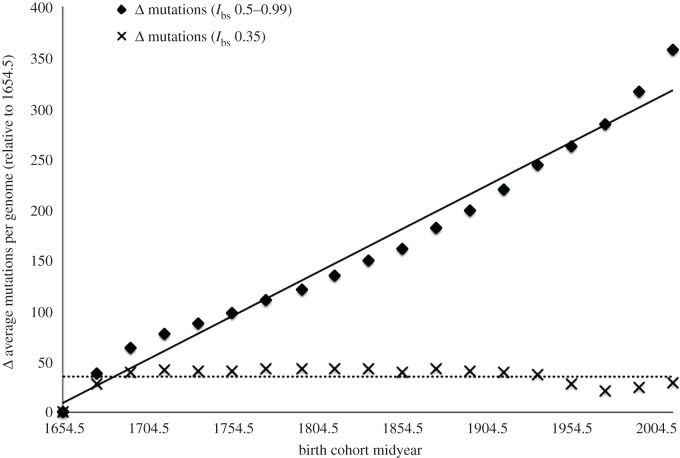
*Δ* average mutations per genome relative to the 1650–1659 birth cohort (the reference sample) under different strengths of negative selection. Temporal correlations of 0.987 (for *I*_bs_ values 0.5–0.99) and −0.003 (for *I*_bs_ value 0.35). Based on reanalysis of Icelandic data reported in [9].

If negative selection purges historical variants at a rate proportional to the observed increase in *I*_bs_, mutation load can be expected to increase at a rate of 16.25 variants every 20 years (*r* = 0.987, 95% CI = 0.966–0.995, *N* = 19 cohorts). Fixing the *I*_bs_ value at 0.35 across cohorts yields no change in mutation load (*r* = −0.003, 95% CI = −0.456–0.451, *N* = 19 cohorts). As with Kong *et al*. [9], unchanging population size and generation lengths are assumed, and fathers are modelled as the sole source of de novo variants. The simulation merely demonstrates that if reduced opportunity for reproductive participation via mortality constitutes the primary source of negative selection in human populations, then such mortality selection needs to be quite strong in order to prevent mutation accumulation—even if mean paternal age at birth is declining.

Arslan *et al*. [1] also mention the possibility that abortions may be a source of negative selection in modernized populations, because they terminate about a fifth of pregnancies. But as those researchers note, most such abortions are elective rather than therapeutic (i.e. they are conducted primarily out of a desire for fewer children). The levels of developmental instability in spontaneously aborted and prematurely deceased babies have been found to be significantly higher than in babies terminated via elective abortions [10]; thus such abortions may not be selective on the whole. It should be noted also that the importance of parental age with respect to mutation load has probably not remained constant over historical time. Importantly, the age of menarche has declined at a rate of two to three months per decade for about a century (nineteenth to twentieth; [11]); studies of more recent samples indicate that the rate of decline of pubertal age is similar in males and females [12]. Therefore, age-matched post-pubescent individuals from different periods may carry different burdens of de novo mutations in their gametes due to differential timing of gametogenesis alone.

Certain types of de novo mutations with potentially significant fitness consequences may be unrelated to paternal age, meaning that this parameter cannot be relied upon to capture all fitness relevant variation due to de novo variants. It has been observed that paternal age is unrelated to de novo copy number variations (CNVs) [13,14]. The apparent lack of a relationship may be due to the fact that structural variants such as CNVs arise via ‘non-allelic homologous recombination during meiosis’ [14, supplementary information]. Structural variants have implications for fitness as they may be significantly related to disorders with severe fitness costs, such as schizophrenia [14].

To comprehensively determine whether mutation loads have increased in modernized populations, it will be necessary to compare genomes from populations that were potentially subjected to different historical strengths of negative selection, such as those from different periods of time or different regions of the world. However, substantial improvements in variant calling precision may be needed to detect these recently accumulated mutations [15]. Nevertheless, consistent with an inference of accumulating deleterious mutations, cross-country analyses have used *I*_bs_ to predict variation in the rates of ‘diseases of modernity’ such as diabetes [16], obesity [17] and cancer [18], net income, lifestyle and other socio-cultural and demographic covariates. There are also indications of secular increases in various minor medical abnormalities, which are suggestive of mutation accumulation [8]. Even if the direct impacts of these variants on human ‘fitness’ in modernized populations are small (e.g. 1% per generation), extrapolated across a century this constitutes a non-trivial decline [19]. Furthermore, just because modernity may have thus far buffered against the effects of reduced negative selection (via advances in medicine and standards of living) does not mean that it can be expected to do so indefinitely [19].

## Data accessibility

Data are publicly available from the Dryad Digital Repository (http://dx.doi.org/10.5061/dryad.kq6cp) [20].

## Authors' contributions

M.A.W.o.M. devised and contributed to writing the comment. M.A.S. contributed to writing the comment. H.B.F.F. produced figure 1.

## Competing interests

No competing interests.

## Funding

We received no funding for this study.
